# Supplementary material for: Selective stimulation of the ferret abdominal vagus nerve with multi-contact nerve cuff electrodes
Source: Sci Rep. 2021 Jun 21;11:12925. doi: 10.1038/s41598-021-91900-1 (PMC8217223; doi:10.1038/s41598-021-91900-1)
Supplement: Supplementary file 1 — Supplementary Figures. [file 41598_2021_91900_MOESM1_ESM.pdf]

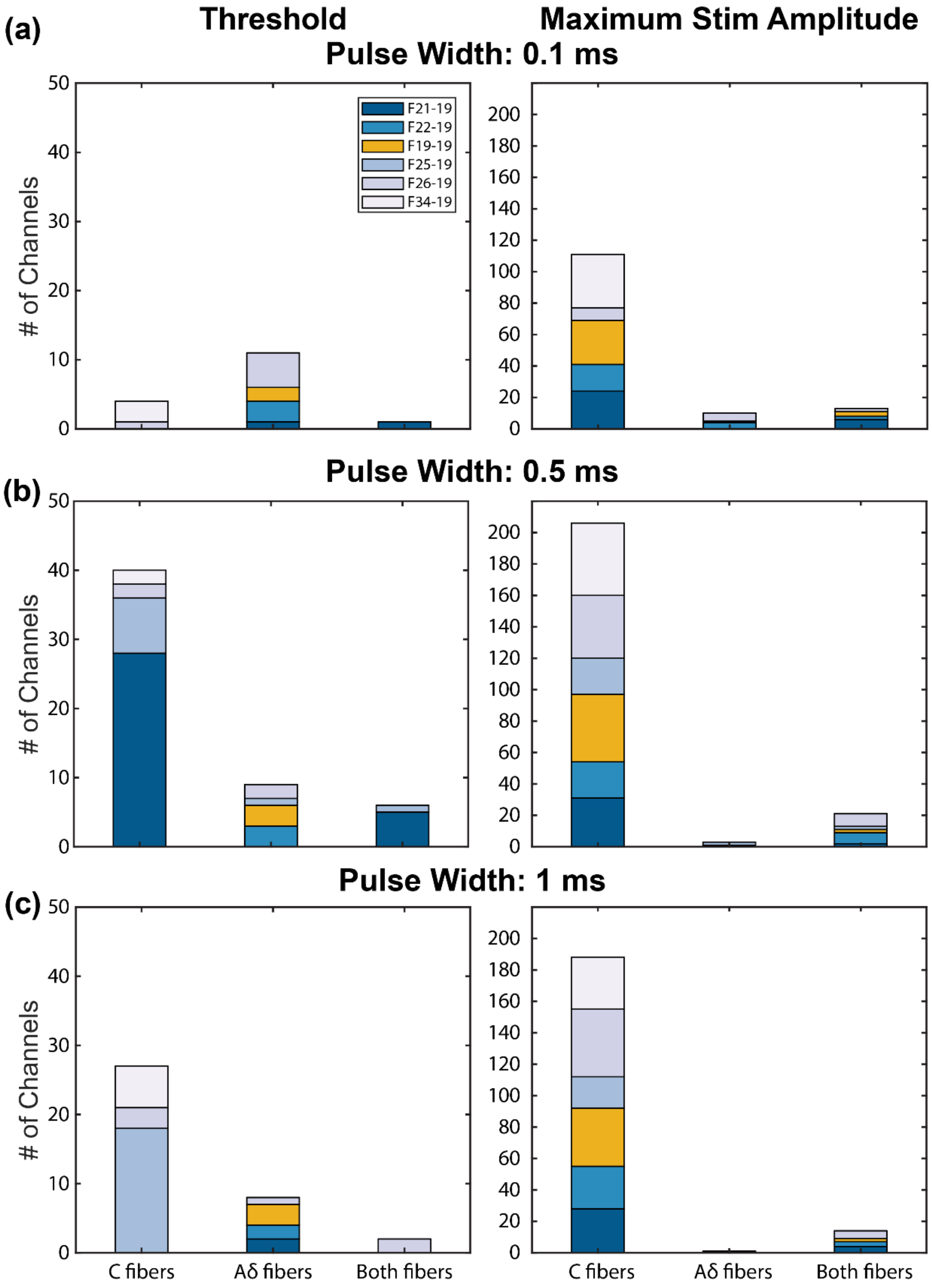

Figure S1. *Fiber types of responses recorded on each MEA channel*. Counts of the number of MEA channels recordin responses corresponding to only C fibers, only Aδ fibers, or both fiber types, at (left) threshold and (right) maximum stimulation amplitude (i.e. 3 mA) for pulse widths of (a) 0.1, (b) 0.5, and (c) 1 ms. With the exception of 0.1 ms pulses, at threshold most MEA channels recorded only c fiber responses, with a smaller set recording Aδ or both responses. At maximum stimulation amplitude, most MEA channels also recorded only c fiber responses, although some channels recorded both fiber types and a small fraction recorded only Aδ responses.

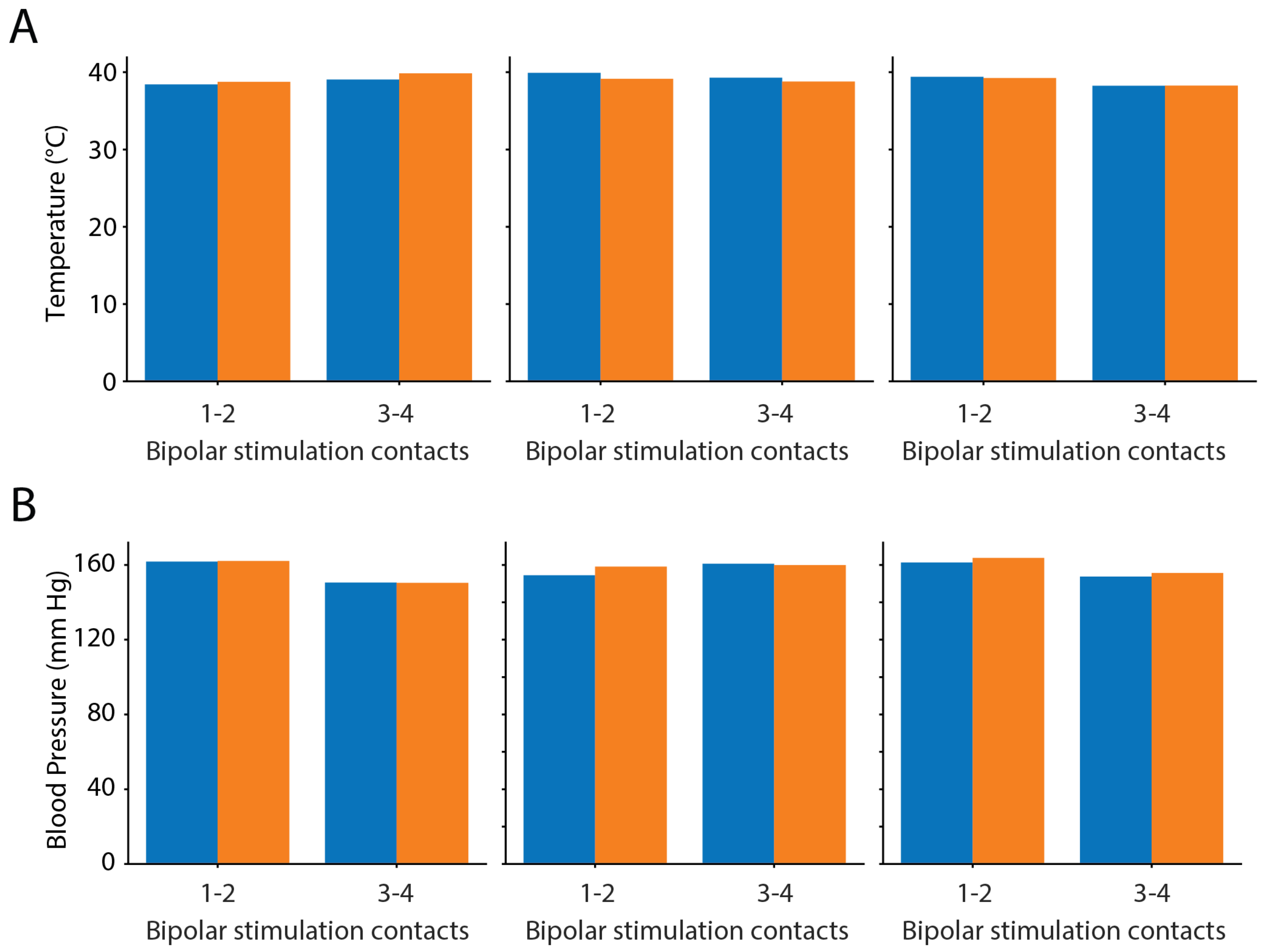

Figure S2. *Effects of vagus nerve stimulation on (a) body temperature and (b) systolic blood pressure in 3 ferrets.* Blue bars represent the average pre-stimulation baseline and orange bars represent the average value during stimulation.
